# Functional Conservation of the Pre-Sensor One Beta-Finger Hairpin (PS1-hp) Structures in Mini-Chromosome Maintenance Proteins of *Saccharomyces cerevisiae* and Archaea

**DOI:** 10.1534/g3.114.011668

**Published:** 2014-05-23

**Authors:** Christopher J. Ramey, Robert A. Sclafani

**Affiliations:** Department of Biochemistry and Molecular Genetics, University of Colorado Anschutz Medical Campus, Aurora, Colorado 80045

**Keywords:** replication, helicase, S phase, cell cycle, yeast

## Abstract

Mini-chromosome maintenance (MCM) proteins form complexes that are required for DNA replication and are highly conserved throughout evolution. The replicative helicase of eukaryotic organisms is composed of the six paralogs *MCM2-7*, which form a heterohexameric ring structure. In contrast, the structure of the archaean replicative MCM helicase is a single Mcm protein that forms a homohexameric complex. Atomic structures of archaeal MCMs have identified multiple beta-finger structures in Mcm proteins whose *in vivo* function is unknown. In the present study, we have investigated the physiological role of the pre-sensor 1 beta**-**hairpin (PS1-hp) beta-fingers of *Saccharomyces cerevisiae* Mcm4p and Mcm5p in DNA replication initiation and elongation *in vivo*. The PS1-hp beta-finger mutant of Mcm5p (*mcm5-HAT K506A*::*URA3*) has a growth defect at both 18° and 37°. Mutation of the Mcm4p PS1-hp beta-finger (*mcm4-HA K658A*::*TRP1*) does not have a growth defect, indicating different functional contributions of the PS1-hp beta-finger structures of different MCM helicase subunits. Both Mcm4p and Mcm5p PS1-hp beta-finger mutants can coimmunoprecipitate Mcm2p, indicating the formation of the hexameric MCM helicase complex. Both PS1-hp beta-finger mutants have a plasmid loss phenotype that is suppressible by origin dosage, indicating a defective replication initiation. Surprisingly, a defect in the binding of PS1-hp MCM mutants to origins of DNA replication was not found by chromatin immunoprecipitation, suggesting a novel interpretation in which the defect is in a subsequent step of DNA strand separation by the MCM helicase. The double mutant *mcm4-HA K658A*::*TRP1mcm5-HAT K506A*::*URA3* is lethal, displaying a terminal MCM mutant phenotype of large budded cells.

DNA replication in eukaryotes is a tightly controlled process. Failure of DNA replication control mechanisms leads to chromosomal instability, mutations, and aneuploidy, which are hallmarks of human disease, including birth defects, aging, and cancer (Jackson *et al.* 2014). The exact duplication of the genome is an essential cellular process. DNA replication control mechanisms ensure that genomic information is replicated exactly once per cell cycle (Sclafani and Holzen 2007). DNA replication begins at DNA sequences termed “origins.” In higher eukaryotes, origins have no defined DNA sequence; however, in *Saccharomyces cerevisiae*, origins of DNA replication have a 14-bp consensus site termed the autonomous replicating sequence (ARS) (Leonard and Mechali 2013; Sclafani and Holzen 2007). Origins are continually bound throughout the cell cycle by the origin recognition complex (ORC), which is a six-subunit ATP-dependent protein complex encoded in yeast by *ORC1-6* (Bell and Dutta 2002; Leonard and Mechali 2013; Sclafani and Holzen 2007).

A two-step process regulates DNA replication. The first step is termed “licensing of the origin,” characterized by the formation of the prereplicative complex (Labib 2010). Licensing consists of loading the mini-chromosome maintenance (MCM) helicase during the G1 phase of the cell cycle onto double-stranded DNA (dsDNA) by Cdt1p and Cdc6p in yeast (Frigola *et al.* 2013; Labib 2010). The MCM helicase is composed of six paralogs *MCM2-7* (Forsburg 2004) and is loaded as a double hexamer (Bell and Botchan 2013; Frigola *et al.* 2013; Gambus *et al.* 2011). Mcm2-7p is thought to be the replicative helicase responsible for DNA stand separation (Bell and Botchan 2013; Forsburg 2004; Tye 1999). The inactive MCM helicase loaded onto the origin of replication along with ORC forms the prereplicative complex. In the second step, the origin of DNA replication is activated in S phase by cyclin-dependent kinase (CDK) and Dbf4-dependent kinase (DDK) (Sclafani and Holzen 2007).

DDK, which is composed of the catalytic Cdc7p and regulatory Dbf4p subunits, phosphorylates the N-terminal tails of Mcm 2/4/6. Phosphorylations are thought to induce structural changes that activate the MCM helicase (Hoang *et al.* 2007; Sclafani and Holzen 2007). It has also been shown that S-phase CDK (S-CDK) phosphorylates the MCM helicase; however, the essential targets of S-CDK are Sld2p and Sld3p (Randell *et al.* 2010; Tanaka *et al.* 2007; Zegerman and Diffley 2007) Phosphorylation of Sld2p and Sld3p allows for the binding of Dpb11p, which leads to the stable association of Cdc45p and the recruitment of the GINs complex and DNA polymerases, activating the origin of DNA replication (Labib 2010). The formation of Cdc45p, GINs, and Mcm2-7p complex is known as the CMG complex and is thought to be the active form of the replicative helicase (Bell and Botchan 2013). The DNA replication origins are activated in a temporal fashion during S phase, with some origins activating (firing) early in S phase, whereas others fire in the middle and later parts of S phase (McCune *et al.* 2008). Temporal control of origin activation is thought to be controlled by DNA replication factor binding mediated by the kinase activities of CDK and DDK.

## MCM Helicase Structure and Function

**Figure 1:**
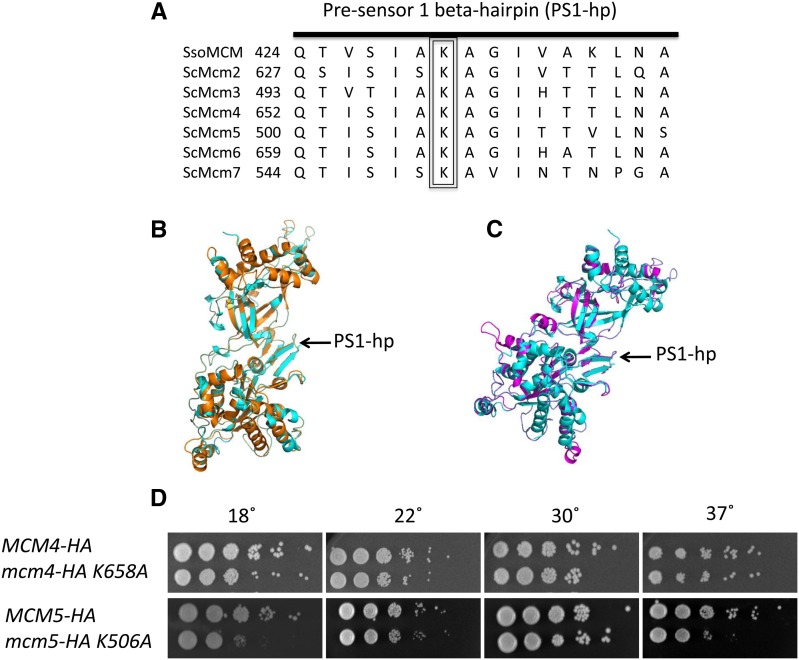
Pre-sensor 1 beta-hairpin structure and growth of mutants. (A) Conservation of the PS1-hp amino acids between *Sulfolobus solfataricus* Mcm and *Saccharomyces cerevisiae* Mcm2-7p. (B) Structure prediction of scMcm4p using Phyre software using the ssoMcm structure as a template. Alignment of the ssoMcm(cyan) and scMcm4p (orange) using pymol software, PS1-hp is indicated with an arrow. (C) Structure prediction of scMcm5p using Phyre software using the ssoMcm structure as a template. Alignment of the ssoMcm(cyan) and scMcm5p (magenta) using pymol software, PS1-hp indicated with an arrow. (D) Serial dilution analysis of growth indicates no growth defect of the Mcm4p PS1-hp mutant compared with wild type (Mcm4p-HA). Yeast with the PS1-hp mutation in Mcm5p (*mcm5-K506A*::*URA3*) has a cold sensitivity phenotype at 18° and a temperature sensitivity phenotype at 37° as indicated by reduced growth compared with the wild type (Mcm5p-HA).

MCM genes are found in both eukaryotes and archaea (Bochman and Schwacha 2009). The eukaryotic MCM helicase is composed of six paralogs *MCM 2-7* that belong to the highly conserved AAA+ ATPase family (Bochman and Schwacha 2009). Mcm2-7p are thought to have diverged from a common ancestor and form a hexameric ring structure with a 1:1:1:1:1:1 stoichiometry (Brewster *et al.* 2008). All Mcm2-7 proteins are required for DNA replication and are essential for viability (Bochman and Schwacha 2009). A single gene encodes the archeal MCMs in *Sulfolobus solfataricus* (*ssoMCM*) and *Methanothermobacter thermautotrophicus* (*mtMCM*) (Brewster *et al.* 2008; Fletcher *et al.* 2003). Analysis of the structures of the N-terminal domain of mtMCM protein and the near full-length structure of ssoMCM protein has revealed interesting beta-hairpin structures of each subunit that are located in the central channel or side channels in the MCM hexamer (Brewster *et al.* 2008; Fletcher *et al.* 2003). Previous work from our laboratory has demonstrated the importance and functional conservation of the N-terminal beta finger in *Saccharomyces cerevisiae*. The N-terminal beta finger of Mcm5p is important for the MCM complex to bind to origins of DNA replication (Leon *et al.* 2008). The pre-sensor 1 beta**-**hairpin (PS1-hp) of MCM complexes is highly conserved (Figure 1) and a mutation in the PS1-hp of ssoMCMp greatly decreases helicase activity on both a 3′-tailed substrate and a Y-shaped substrate *in vitro* (McGeoch *et al.* 2005). The PS1-hp is thought to have nucleotide dependent movement that promotes helicase activity (Bell and Botchan 2013; Slaymaker and Chen 2012). The PS1-hp is conserved in the distantly related Superfamily 3 helicases, which includes SV40 T antigen, and has been shown to undergo significant movement during nucleotide binding, hydrolysis, and release cycle of the helicase (Bell and Botchan 2013; Gai *et al.* 2004; Slaymaker and Chen 2012). In this current study, we present a molecular genetic analysis of mutations of the highly conserved lysine residue of the PS1-hp in *Saccharomyces cerevisiae* Mcm4p and Mcm5p (Figure 1).

## Materials and Methods

### Yeast strains, media, and plasmids

Yeast strains are listed in Table 1 and plasmids are listed in Table 2. Yeast strains were grown using standard procedures as well as methods published previously (Leon *et al.* 2008). To produce integrating plasmids carrying *mcm4-HA K506A* and *mcm5-HA K506A*, Quickchange mutagenesis (Stratagene) was used on plasmid pRS663 (*MCM4-HA*) and pRPL104 (*MCM5-HAT*) and mutations were marked by the addition of restriction sites *Pst*I (*MCM4-HA*) and *Pvu*II (*MCM5-HAT*). All mutations were verified by polymerase chain reaction (PCR), restriction digest, and by sequencing. Yeast strains were constructed using a plasmid shuffle strategy using a covering wild-type (WT) gene on a plasmid. Plasmids carrying either *MCM4-HA* (pRS663) or *mcm4-HA K658A* (pCJR101) were digested with *Nru*I and integrated at the endogenous *MCM4* locus, creating a partial duplication with the active copy containing a hemagglutinin (HA) tag or HA tag and the PS1-hp mutation. *MCM5-HA* or *mcm5-HA K506A* was constructed using pRPL104 (*MCM5-HAT*) or pRS734 (*mcm5-K506A-HAT*) digested with PpuMI to integrate at the ura3-1 locus. *MCM5* at the endogenous locus was deleted with *KanMX4* and covered with a WT *MCM5* plasmid (pRS660) that was shuffled with the tagged version and the tagged PS1-hp mutant version to test for viability.

**Table 1:** *S. cerevisiae* strains used

Yeast Strain	Genotype	Source
RSY311	*MAT****a*** *trp1 leu2 ura3 can1 his6 bar1*	(Leon *et al.* 2008)
CRY106	*MAT****a*** *trp1 leu2 ura3 can1 his6 bar1 MCM4-HA*::*TRP1*	This study
CRY107	*MAT****a*** *trp1 leu2 ura3 can1 his6 bar1 mcm4-HA K658A*::*TRP1*	This study
CRY109	*MATα trp1 leu2 ura3 can1 his6 bar1 mcm4-HA K658A*::*TRP1*	This study
CRY111	*MAT****a*** *trp1 leu2 ura3 can1 his6 bar1 MCM4-HA*::*TRP1[ pDK243]*	This study
CRY112	*MAT****a*** *trp1 leu2 ura3 can1 his6 bar1 MCM4-HA*::*TRP1 [pDK368-7]*	This study
CRY113	*MAT****a*** *trp1 leu2 ura3 can1 his6 bar1 mcm4-HA K658A*::*TRP1[ pDK243]*	This study
CRY114	*MAT****a*** *trp1 leu2 ura3 can1 his6 bar1 mcm4-HA K658A*::*TRP1[ pDK368-7]*	This study
CRY119	*MATα trp1 leu2 ura3 can1 his6 bar1 mcm4-HA K658A*::*TRP1*	This study
CRY175	*MAT****a*** *ura3*::*MCM5-HAT URA3 tyr1 leu2 ade1 ade2 mcm5∆*::*kanMX4 trp1 cyh2 [pDK243]*	This study
CRY176	*MAT****a*** *ura3*::*MCM5-HAT URA3 tyr1 leu2 ade1 ade2 mcm5∆*::*kanMX4 trp1 cyh2 [pDK368-7]*	This study
CRY177	*MAT**a** ura3*::*mcm5-K506A-HAT URA3 tyr1 leu2 ade1 ade2 mcm5∆*::*kanMX4 trp1 cyh2 [pDK243]*	This study
CRY178	*MAT****a*** *ura3*::*mcm5-K506A-HAT URA3 tyr1 leu2 ade1 ade2 mcm5∆*::*kanMX4 trp1 cyh2 [pDK368-7]*	This study
CRY210	*MAT****a*** *ura3 tyr1 leu2 ade1 ade2 mcm5∆*::*kanMX4 trp1 cyh2 [pRS660 MCM5*::*LEU2]*	This study
CRY211	*MAT****a*** *ura3*::*mcm5-K506A-HAT URA3 tyr1 leu2 ade1 ade2 mcm5∆*::*kanMX4 trp1 cyh2 [pRS660 MCM5*::*LEU2]*	This study
CRY212	*MAT****a*** *ura3*::*mcm5-K506A-HAT URA3 tyr1 leu2 ade1 ade2 mcm5∆*::*kanMX4 trp1 cyh2 mcm4-K658A-HA*::*TRP1 [pRS660 MCM5*::*LEU2]*	This study
RSY1259	*MAT****a*** *ura3 tyr1 leu2 ade1 ade2 mcm5∆*::*kanMX4 trp1 cyh2 [ pRS414 MCM5*::*TRP1]*	(Leon *et al.* 2008)
RSY1336	*MAT****a*** *ura3*::*URA3 MCM5-HAT tyr1 leu2 ade1 ade2 mcm5∆*::*kanMX4 trp1 cyh2 bar1*::*LEU2*	(Leon *et al.* 2008)
RSY1345	*MAT****a*** *ura3*::*URA3 mcm5-K506A-HAT tyr1 leu2 ade1 ade2 mcm5∆*::*kanMX4 trp1 cyh2 bar1*::*LEU2*	(Leon *et al.* 2008)
RSY1148	*MAT****a*** *trp1 leu2 ura3 can1 his6 bar1 mcm5-461*	(Leon *et al.* 2008)
CRY205	*MAT****a*** *trp1 leu2 ura3*::*URA3 MCM5-HAT can1 his6 bar1 mcm5-461*	This study
CRY206	*MAT****a*** *trp1 leu2 ura3*::*URA3 mcm5-K506A-HAT can1 his6 bar1 mcm5-461*	This study
CRY207	*MAT****a*** *trp1 leu2 ura3*::*URA3 MCM5-HAT can1 his6 bar1 mcm5-461 mcm4-K658A-HA*::*TRP1*	This study
CRY208	*MAT****a*** *trp1 leu2 ura3*::*URA3 mcm5-K506A-HAT can1 his6 bar1 mcm5-461 MCM4-HA*::*TRP1*	This study
CRY209	*MAT****a*** *trp1 leu2 ura3*::*URA3 mcm5-K506A-HAT can1 his6 bar1 mcm5-461 mcm4-K658A-HA*::*TRP1*	This study
CRY213	*MAT****a*** *ura3*::*URA3 MCM5-HAT tyr1 leu2 ade1 ade2 mcm5∆*::*kanMX4 trp1 cyh2*	This study
CRY214	*MAT****a*** *ura3*::*URA3 mcm5-K506A-HAT tyr1 leu2 ade1 ade2 mcm5∆*::*kanMX4 trp1 cyh2*	This study

**Table 2:** Recombinant plasmids used

Plasmid Name	Genotype	Source
pRAS660	pRS305 ARS CEN *LEU2 MCM5*	(Leon *et al.* 2008)
pRAS662	pRS305 ARS CEN *LEU2 MCM4*	(Leon *et al.* 2008)
pRPL104	pRS306 ARS CEN *URA3 MCM5-HAT*	(Leon *et al.* 2008)
pDK243	ARS(1x) CEN *LEU2*	(Hogan and Koshland 1992)
pDK368-7	ARS(8X) CEN *LEU2*	(Hogan and Koshland 1992)
pRAS663	pRS404 *mcm4-HA*	(Aparicio *et al.* 1999)
pCJR101	pRS404 *mcm4-K658A-HA*	This study
pRAS734	pRS306 *mcm5-K506A-HAT*	This study

### Plasmid stability assay

To generate the strains used for plasmid loss assays, CRY106, CRY107, CRY213, and CRY214 were transformed with either pDK243 *LEU2* (1X-ARS site) or pDK368-7 *LEU2* (8X-ARS sites), and Leu^+^ transformants were selected generating strains. WT and PS1-hp mutant strains containing either pDK243 *LEU2* (1X-ARS site) or pDK368-7 (8X-ARS sites) with a *LEU2* marker were grown in synthetic complete media–leucine to stationary phase, then plated onto YEPD and synthetic complete media–leucine to determine plasmid stability under selection. Stationary cultures were then diluted to 2 × 10^3^ cells/mL and grown in synthetic complete media + leucine (relaxed conditions) and allowed to grow to stationary phase. Stationary cultures were then plated onto yeast extract peptone dextrose and synthetic complete media – leucine to determine plasmid stability under relaxed selection. The plasmid loss rate was determined by the observed loss of the plasmid on synthetic complete–leucine plates compared with yeast extract peptone dextrose plates under relaxed selection as described previously (Hogan and Koshland 1992; Leon *et al.* 2008). At least three independent loss rates were calculated for each condition.

#### Co-immunoprecipitation (IP) and western blot analysis:

Protein extracts were prepared as described previously (Leon *et al.* 2008). In summary, 2 mg of protein extract were incubated with 30 μL of protein G-Sepharose beads (Sigma-Aldrich) blocked in lysis buffer supplemented with complete protease inhibitor tablets from Roche (cat. no. 05892970001) for 1 hr at 4°. IP was performed using 50 μg of anti-HA antibody (Roche). Negative controls were performed without antibody, containing only protein G-sepharose beads, and all reactions were incubated with end-over-end rotation at 4° for 3 hr. Samples were then centrifuged at 240*g* at 4° and the supernatant was collected and mixed with 5X sodium dodecyl sulfate (SDS) buffer to a final of 1X and boiled for 5 min. Pellets were washed three times in 500 μL of lysis buffer containing complete protease inhibitors tablets from Roche (cat. no. 05892970001) and were then resuspended in 50 μL of 2.5X SDS buffer. Western blot analysis used 10% of each fraction, whole-cell extract, supernatant, and pellet. Samples of IPs were resolved on a 7% SDS-polyacrylamide gel electrophoresis gel, transferred to a nitrocellulose membrane, and probed with anti-HA antibody (Roche) at a 1:2500 dilution, anti-Mcm2p antibody (Santa Cruz) at a 1:1000 dilution, and anti-Cdt1p (UM185) at a 1:15000 dilution. For HA blots, a secondary antimouse antibody conjugated to horseradish peroxidase (HRP; Jackson ImmunoResearch) was used at 1:3000 dilution. For Mcm2p blots, a secondary antigoat antibody conjugated to HRP (Santa Cruz Biotechnology) was used at a 1:3000 dilution. For Cdt1p blots, a secondary antirabbit antibody conjugated to HRP (Bio-Rad) was used at a 1:3000 dilution. Immunoblots were visualized on film or using a ChemiDoc MP scanner (Bio-Rad) employing an ECL chemiluminescence kit (Millipore).

#### Chromatin immunoprecipitation (ChIP) analysis:

ChIP analysis was performed as previously described (Adkins *et al.* 2004) with the following modifications. Forty-five milliliters of alpha factor G1 arrested yeast cultures (2 × 10^7^ cells/mL) were fixed with formaldehyde for 1 hr at room temperature. Cells were lysed using a mini bead buster (Biospec) for 3X 2-min intervals in ChIP lysis buffer 1 [50 mM HEPES, pH 7.5; 140 mM NaCl; 1 mM ethylenediaminetetraacetic acid (EDTA); 1% Triton X-100; 0.1% deoxychloic acid; complete protease inhibitor tablet (Roche cat nol. 05892970001)]. Cell lysis was sonicated using a Bioruptor (Diagenode) for 30 cycles of 30 sec on/off at 4°. Cell debris was removed with two 20,000*g* spins for 5 min. The chromatin fraction was precleared with the addition of 10 μL of Protein G sepharose beads (Sigma-Aldrich) with end-over-end rotation for 1 hr followed by centrifugation at 880*g* for 1 min. Then, 50 μL of cleared lysate was taken as an input sample with the addition of 30 μL of ChIP elution buffer (50 mM Tris-HCl, pH 7.5; 10mM EDTA; 1% SDS). IPs were set up with 350 μL of chromatin solution per IP reaction. For HA IPs, the chromatin fraction was incubated with 25 μg of ant-HA antibody (Roche). All IPs were incubated for 15 hr at 4°. Protein G sepharose beads (30 μL) were added to each reaction for 1 hr at 4°. Beads were washed 2X with ChIP lysis buffer 1 (50 mM HEPES, pH 7.5; 140mM NaCl; 1 mM EDTA; 1% Triton X-100; 0.1% deoxycholic acid), twice with lysis buffer 2 (50 mM HEPES, pH 7.5; 500 mM NaCl; 1 mM EDTA; 1% Triton X-100; 0.1% deoxycholic acid), twice with lysis buffer 3 (10 mM Tris, pH 7.9; 250 mM LiCl; 1 mM EDTA; 0.5% NP-50; 0.5% deoxycholic acid), and once with TE. Beads were resuspended in 80 μL of ChIP elution buffer with 20 μL of pronase at 20 mg/mL (Calbiochem), incubated at 42° for 2 hr, followed by 8 hr at 65° to reverse the crosslinks. Chromatin-associated DNA isolated by ChIP was subjected to quantitative PCR, using 2X Sybergreen master mix from (Roche) and a Roche Lightcycler 480 thermocycler. Chromatin associated DNA was subjected to PCR analysis with primers to the following DNA replication origins: *ARS1*, *ARS305*, *ARS306*, *ARS603*, *ARS607*, and *ARS1412*.

### Determining whether Mcm4p Mcm5p PS1-hp double mutant requires a WT Mcm5p encoding plasmid for viability

Yeast strain CRY214 (*mcm5-HA K506A*::*URA3*) was transformed with pRS660 (WT *MCM5*). Next, *mcm4-HA K658A*::*TRP* was created by integration and partial duplication at the *MCM4* locus. To test whether the viability of the Mcm4p Mcm5p PS1-hp double mutants requires the WT Mcm5p encoding plasmid, the loss rate of the WT *MCM5* plasmid under relaxed conditions was compared between the double PS1-hp mutant and the single mutant *mcm5-HA K658A*::*URA3*.

#### Lethality of the Mcm4p Mcm5p PS1-hp double mutant by tetrad analysis:

CRY119 (*MATα mcm4-HA K658A*::*TRP1*) was mated to RSY1345 (*MAT***a** *mcm5-HA K506A*::*URA3* integrated at *ura3-1 mcm5*::*KanMX4*) (Table 1). Diploids were selected on synthetic complete –TRP –URA plates and sporulated in 3% potassium acetate. Ninety-one tetrads were dissected, and spores were germinated on YPD plates at 30°. Spores that were Ura^+^ G418R were tested for the Trp^+^ phenotype, indicating double mutant (*mcm4-HA K658A*::*TRP1mcm5-HA K658A*::*URA3mcm5*::*KanMX4*) viability. The terminal phenotype was determined by photographing spores know to be a double PS1-hp mutants.

## Results

### PS1-hp mutations of *Mcm4* and *Mcm5* are viable

The PS1-hp beta-finger structure is conserved in both Mcm4 and Mcm5 proteins of yeast by both primary sequence and structural alignment (Figure 1, A−C). Importantly, a conserved lysine is present at the tip of the beta-finger in both proteins. Yeast strains with integrated copies of the lysine (K) to alanine (A) PS1-hp mutations in Mcm5p and Mcm4p were constructed to produce *mcm4 K658A*::*TRP1* and *mcm5 K506A*::*URA3* strains CRY107 and RSY1345, respectively, as described in the *Materials and Methods*. These strains have a PS1-hp mutant *mcm4* or *mcm5* gene as the only copy of the gene in the cell, under the control of its native promoter. Because both temperature-sensitive and cold-sensitive *mcm* mutants have been described previously (Tye 1999), we tested for growth at a range of temperatures. Ten-fold serial-dilution analysis of the *mcm5 K506A*::*URA3* strain indicates both a temperature-sensitive phenotype and a cold-sensitive phenotype, with reduced growth compared with the *MCM4 or MCM5* wt strain at 37° and 18° (Figure 1D). In contrast, the corresponding PS1-hp mutation on Mcm4p (*mcm4 K658A*::*TRP1*) does not display a temperature-sensitive or cold-sensitive phenotype (Figure 1D). To test for sensitivity of PS1-hp mutants to replication stress, 10-fold serial-dilution analysis of growth was performed on plates containing different amounts of hydroxyurea, but no hydroxyurea sensitivity was found (Supporting Information, Figure S2) indicating that the checkpoint is intact in PS1-hp mutants. Introduction of the PS1-hp mutation into Mcm4p or Mcm5p does not have an effect on protein stability and both mutant proteins are expressed from their own promoters (Figure S1).

### The Mcm4p and Mcm5p PS1-hp mutant proteins are incorporated into the Mcm2-7p complex

**Figure 2:**
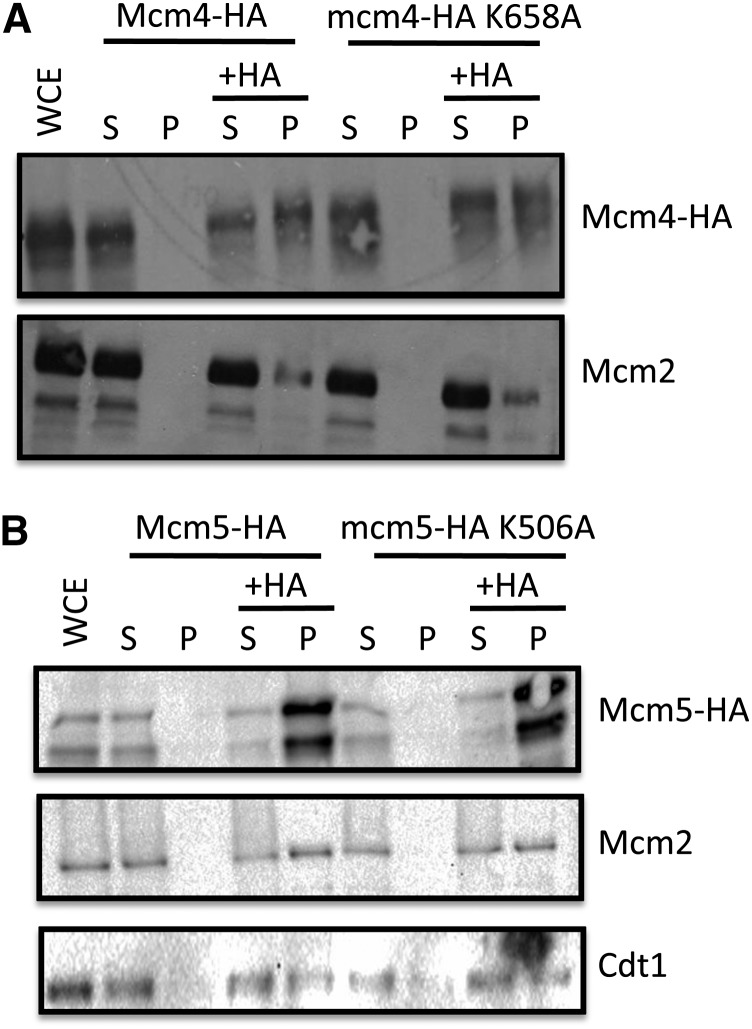
Co-immunoprecipitation of the Mcm4p and Mcm5p PS1-hp mutants. (A) Immunoprecipitations of Mcm4p-HA, mcm4p-HA K658A, Mcm5p-HA, and mcm5p-HA K506A were performed using an anti-HA antibody or a no antibody negative control. Western blots were probed with anti-HA antibody for Mcm5p, an anti-Mcm2p antibody, and an anti-Ctd1p antibody. Supernatant and pellet fractions are represented by “S” and “P,” respectively. HA, hemagglutinin.

To demonstrate that the PS1-hp mutants get incorporated into the Mcm2-7p hexamer, both WT and PS1-hp mutants of both Mcm4p and Mcm5p were tagged with an HA epitope. An anti-HA antibody was used to precipitate each of the following: Mcm4p-HA, mcm4p-HA K658A, Mcm5p-HA, and mcm5p-HA K658A. Immunoprecipitates were run on 7.5% SDS-polyacrylamide gel electrophoresis gels, transferred to nitrocellulose, and probed for Mcm2p using an anti-Mcm2p antibody to demonstrate Mcm2-7p complex formation. Mcm2p binds directly to Mcm5p and Mcm6p and only binds indirectly with Mcm4p in the hexameric MCM ring. Immunoprecipitates from both Mcm4p-HA and mcm4p-HA K658A strains contain similar amounts of Mcm2p, indicating that the PS1-hp of Mcm4p forms the hexameric MCM complex *in vivo* (Figure 2A). Similar to Mcm4p, both Mcm5p-HA and mcm5p-HA K506A immunoprecipitates contain equal amounts of Mcm2p, demonstrating Mcm5p binds Mcm2p in the Mcm5p PS1-hp mutant (Figure 2B.). In addition to Mcm2p, mcm5p-HA K506A co-immunoprecipitates with Cdt1p at WT levels (Figure 2B), indicating that Mcm5p PS1-hp can interact with Cdt1p, which is important for MCM helicase loading at origins of DNA replication (Tanaka and Diffley 2002). Because Cdt1p is known to interact with the hexameric MCM helicase, these data indicate Mcm5p PS1-hp mutant can form the MCM complex. These results are consistent with results found *in vitro* when analyzing the MCM complex formation of the PS1-hp mutants of SsoMCM (McGeoch *et al.* 2005).

### Plasmid loss phenotype of Mcm4p and Mcm5p PS1-hp mutants

**Figure 3:**
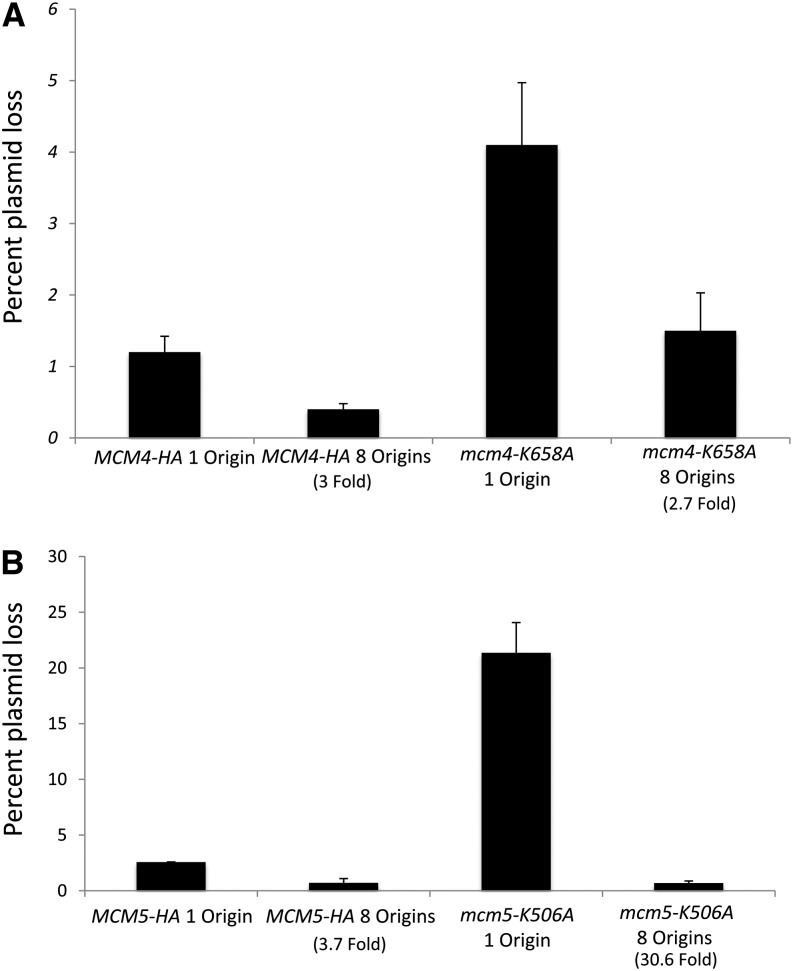
Plasmid stability of Mcm4p and Mcm5p PS1-hp mutants. (A) Wild type or *mcm4-HA K658A*::*TRP1* or (B) wild type or *mcm5-HAT K506A*::*URA3* yeast strains were transformed with either pDK243 (1 origin of replication) or pDK368-7 (eight origins of replication), and plasmid stability was analyzed. Plasmid loss rates are shown in % loss per generation. The increase in stability with the addition of seven origins of replication is presented as fold increase in stability compared with one origin of replication.

Yeast strains containing the *mcm4-K658A* PS1-hp mutation have a plasmid loss rate per generation of 4.1%, which is threefold higher than the loss rate of 1.2% per generation in wt cells (Figure 3A). To test whether the plasmid loss was due to initiation of DNA replication or elongation, we examined the plasmid loss rate with a plasmid carrying eight origins (Hogan and Koshland 1992). The plasmid loss rate was decreased 2.5-fold in a Mcm4p-PS1-hp mutant compared to a 3-fold reduction in wt with the addition of seven ARS elements, consistent with a defect in replication initiation (Figure 3A). This result was unexpected and inconsistent with the published elongation defect seen for the ssoMCM PS1-hp mutant *in vitro* (McGeoch *et al.* 2005). Next, we examined the plasmid loss rate of the PS1-hp mutant of Mcm5p. The *mcm5-HA K506A*::*URA3* PS1-hp mutant has an increased plasmid loss rate of 21.4%, which is 8-fold greater than the loss rate of 2.6% in wt cells. With the addition of seven origins, the loss rate was reduced about 30-fold for *mcm5-k506A*::*URA3* compared with 3.5-fold in wt cells(Figure 3B). Thus, both *mcm4* and *mcm5* PS1-hp mutants had an increase in plasmid loss with a clear MCM phenotype that was partially suppressible by increased origin dosage. To further investigate this phenotype, we used ChIP analysis to determine Mcm2-7p helicase binding to chromatin at cellular origins of DNA replication.

**Figure 4:**
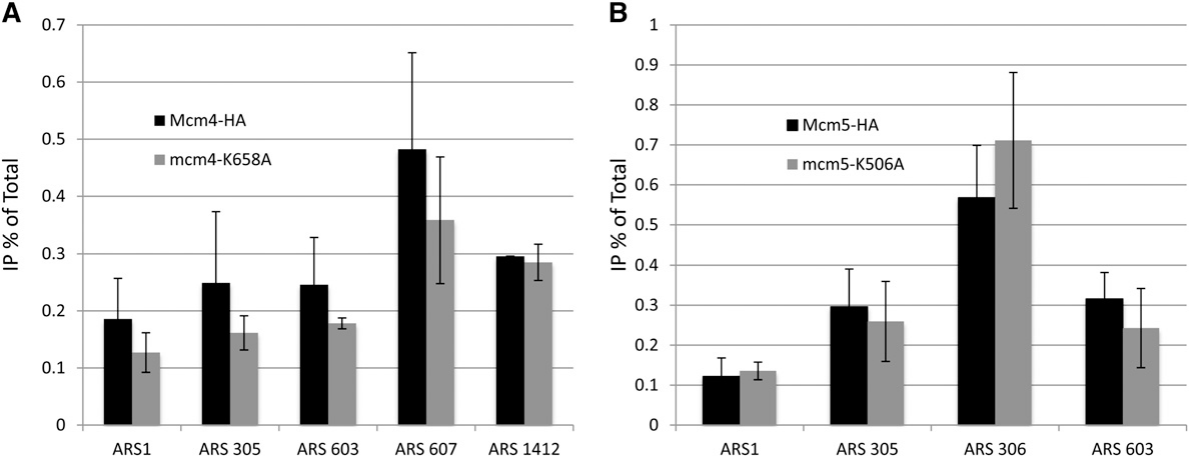
Chromatin immunoprecipitation (ChIP) analysis of Mcm4p and Mcm5p PS1-hp mutant proteins at origins of replication. (A) ChIP analysis of wild-type (*Mcm4-HA*) and PS1-hp mutant of Mcm4p (*mcm4-K658A-HA*) from G1-arrested cultures. Log-phase cultures were arrested in G1 with the addition of alpha factor for 3 hr and an anti-HA antibody was used for ChIP. Chromatin associated DNA was analyzed using quantitative polymerase chain reaction using primers specific to origins of DNA replication. (B) Similar ChIP analysis of wild type (*Mcm5-HA*) and PS1-hp mutant Mcm5p (*mcm5-K506A-HA*) strains.

We used cells arrested in G1 phase of the cell cycle where Mcm2-7 protein binding to origin chromatin is maximal (Aparicio *et al.* 1999). Different origins were examined using ChIP to gain a broad understanding of the affects of the PS1-hp mutation in MCM complex binding to origins of DNA replication. We found no significant defect in origin binding by ChIP of MCM complexes containing the Mcm4p PS1-hp mutation at all 5 origins examined (Figure 4A), which includes early (*ARS305*, *ARS607*), mid (*ARS1 = ARS416*), and late (*ARS603*, *1412*) origins (Raghuraman *et al.* 2001). Analysis of Mcm5p PS1-hp mutants binding to four origins of DNA replication also showed no binding defects detectable by ChIP analysis in G1 arrested cultures (Figure 4B).

To gain further insight into the plasmid loss defect of PS1-hp mutants, a conditional Mcm4p PS1-hp and a Mcm5p PS1-hp double mutant was constructed in a background containing the temperature-sensitive allele *mcm5-461* (Dalton and Hopwood 1997; Leon *et al.* 2008). At 37° the Mcm5-461 protein is unable to integrate into the Mcm2-7p complex (Dalton and Hopwood 1997; Leon *et al.* 2008), creating a double mcm4p mcm5p PS1-hp mutant cell. The following strains were used to observe S-phase progression by flow cytometry of the PS1-hp double mutant: RSY1148 (*mcm5-461*) and CRY209 (*mcm5-461 mcm5-HAT K506A*::*URA3mcm4-HA K658A*::*TRP1*). Yeast strains were arrested in alpha factor for 3 hr at 22° then raised to 37° in alpha factor for 1 hr, then released into S-phase at 37° by the addition of pronase. The resolution of this flow cytometry experimental approach was unable to determine if PS1-hp double mutants progressed more slowly through S-phase when compared to either single PS1-hp mutant alone (Figure S3). The *mcm5-461* temperature sensitive mutant is leaky because eventually cells progress through the cell cycle and will form colonies at 37° after several days (Leon *et al.* 2008). Therefore, we produced double *mcm4-HA K658A*::*TRP1mcm5-HAT K506A*::*URA3* mutants using either plasmid shuffling or yeast meiotic recombination techniques.

### Mutation of the PS1-hp in both mcm4 and mcm5 is lethal

We tested the viability of a *mcm4-HA K658A*::*TRP1mcm5-HA K506A*::*URA3* double mutant in two different ways. Yeast strains with an integrated *mcm5-HA K506A*::*URA3* mutation and a WT 2μ LEU2 *MCM5* plasmid can easily lose the plasmid in that >99% of the cells had lost the plasmid (n = 1836 colonies) after growth in nonselective media for >10 doublings. In contrast, the double *mcm4-HA K658A*::*TRP1mcm5-HA K506A*::*URA3* mutant maintained the plasmid 100% of the time in that 0% of the cells (n = 2039 colonies; *P* < 0.0025) had lost the plasmid under similar conditions, indicating a requirement of the plasmid *MCM5* gene for viability.

Because the *MCM5* gene was on plasmid pRAS690, it is possible that only the plasmid with a single ARS is unable to replicate as was found for the *mcm5-bob1* mutation in the absence of Cdc7p function (Hoang *et al.* 2007). This creates an artificial situation in which the cells do not divide only because the essential plasmid, but not the genome, is unable to replicate. To confirm the synthetic lethality of the two PS1-hp mutants when both are integrated in the genome, CRY119 (*mcm4-HA K658A*::*TRP1* integrated as a partial duplication at the *mcm4* locus) was mated to RSY1345 (*mcm5-HA K658A*::*URA3 at the ura3-1 locus*) that also had a *mcm5*::*KanMX4* deletion to form diploids, which were then sporulated. Tetrads were dissected and spores that were Ura^+^ G418R, which contain both the *mcm5*::*KanMX4 and mcm5-HA K658A*::*URA3* mutations, were tested for the presence of the *mcm4-HA K658A*::*TRP1* allele. As *mcm5* and *mcm4* are unlinked, we expected to find 50% Trp^+^ colonies that carry the *mcm4* PS1-hp mutation. With *mcm5-HA K658A*::*URA3*, no Trp^+^ colonies were found (n = 44, *P* < 0.005) (Figure S4A). Thus, the double *mcm4mcm5* PS1-hp mutant is inviable in cells with the normal complement of cellular origins. The terminal phenotype of the germinated spore of a double *mcm4mcm5* PS1-hp mutant is two large budded cells indicative of a helicase defect as originally described for *mcm2∆* and *mcm3∆* mutants (Figure S4B)(Yan *et al.* 1991).

## Discussion

Using the archaeal structure of MCM as a model, we have confirmed the role of the SsoMCM PS1-hp structure that had been examined in *in vitro* experiments (McGeoch *et al.* 2005) as also being important for downstream events after binding of DNA origins *in vivo* using the model system *Saccharomyces cerevisiae*. This is further *in vivo* evidence that structural models of the homohexameric structures of archaeal MCMs are a valuable tool in understanding the function of eukaryotic heterohexameric MCM complexes (Brewster *et al.* 2008; Fletcher *et al.* 2003; Leon *et al.* 2008). Our results demonstrate that *in vivo* yeast PS1-hp mutants can form the heterohexameric MCM complex and bind origins of DNA replication, but fail to maintain plasmid stability, indicating a defect in the transition to an active MCM complex, or an MCM complex that is defective in efficient strand separation during replication elongation.

The MCM helicase has been clearly shown to load onto dsDNA in the G1 phase of the cell cycle (Leon *et al.* 2008; Remus *et al.* 2009). Our laboratory has previously shown the importance of N-terminal beta-fingers for the MCM complex to bind dsDNA (Leon *et al.* 2008). The circular MCM complex is loaded onto dsDNA using a break in the MCM ring between Mcm5p and Mcm2p (Bochman and Schwacha 2007) by the actions of Ctd1 and Cdc6p (Randell *et al.* 2006; Remus *et al.* 2009). One model of DNA strand separation by the MCM helicase is that of strand exclusion, in which the leading strand DNA remains in the central cavity of the complex bound to the β-hairpins, and the lagging strand is extruded and bound to the PS1 hairpin (Bell and Botchan 2013; Bochman and Schwacha 2009; Fu *et al.* 2011; Slaymaker and Chen 2012). One possible defect of Mcm PS1-hp mutants is the inability to undergo the transformation of binding dsDNA in the inactive form to excluding one strand of DNA in the active form, which would be consistent with our plasmid loss and ChIP results. During the review of this paper, we became aware of a new publication that demonstrates a PS1-hp mutation in yeast Mcm3p is lethal, although the MCM complex with the mutant Mcm3p is properly formed as interpreted from gel filtration experiments (Lam *et al.* 2013)

We found that MCM helicase PS1-hp mutants bind origins of DNA replication at normal occupancy levels and propose PS1-hp mutants are defective in a transition step, which is recovered by the addition of multiple origins by mass action. In plasmid loss experiments, only mutants in initiation such as in *cdc6* (Hogan and Koshland 1992) or *orc2* (Loo *et al.* 1995) are suppressed by increased origin gene dosage. Similarly, *mcm5* mutants in the N-terminal β-hairpins have a similar phenotype and displayed reduced binding to origins by ChIP (Leon *et al.* 2008). In contrast, plasmid loss rates are not suppressed in *pol1* mutants, which are defective in elongation by DNA polymerase α (Hogan and Koshland 1992). We propose that the Mcm PS1-hp mutant proteins can bind to origins normally but have increased dissociation due to a reduced ability to bind the extruded lagging strand. In support of this idea, the DNA helicase activity and the single-stranded DNA (ssDNA) binding stability of recombinant MCM helicases in an electromobility shift assay *in vitro* was lower for MCM2-7 complexes containing a PS1-hp mutation in Mcm3p, possibly because the ssDNA-MCM complexes formed are unstable (Lam *et al.* 2013)

In this scenario, this dissociation defect is suppressed by having more Mcm2-7p complexes at more origins with only one active helicase still bound to complete replication. Our study suggests a different interpretation of the plasmid stability assay when considering mutants of the Mcm helicase or of other proteins that load in G1 phase and are activated in S phase. This leads to a new understanding of the plasmid stability assay, such that multiple origins not only recover binding defects but also defects in transition from an inactive helicase form to an active replicative form.

We found that the *mcm5* and *mcm4* PS1-hp mutants behave differently in that the *mcm5* mutant was more deleterious with a higher plasmid loss defect (Figure 3) and displays both temperature-sensitive and cold-sensitive conditional phenotypes (Figure 1). This is similar to our previous analysis with the β-hairpin mutants (Leon *et al.* 2008). Perhaps, the Mcm5 protein is more important in stabilizing the channel in ssDNA strand exclusion.

Also similar to our previous studies (Leon *et al.* 2008), lethality required mutations in two different *MCM* genes, which we propose reflects the fact that the six PS1-hp structures in the heterohexamer must cooperate for function and do not act independently, as also has been found for mutations in the ATPase site (Schwacha and Bell 2001). It is also possible that lethality results because the Mcm4 and Mcm5 proteins lie opposite each other in the hexameric ring (Bell and Botchan 2013). In the recent study cited previously (Lam *et al.* 2013) all combinations of the other MCM helicase PS1-hp mutations (*MCM2*, *4*, *5*, *6*, and *7*) were lethal except for the *mcm4-K568A* and *mcm5-K506A* combination, which had a slow growth phenotype. The later result contradicts our findings that the *mcm4-K568A* and *mcm5-K506A* combination is lethal. Furthermore, our *mcm5-K506A* mutant has a conditional phenotype (Figure 1), while theirs had no phenotype. One possible explanation is we used a different yeast strain background (A364a) compared to them (S288C). Nonetheless, our proposal in which important structures in MCM subunits cooperate and have similar functions is supported by both studies.

## Supplementary Material

Supporting Information
