# Supplementary material for: Functional Conservation of the Pre-Sensor One Beta-Finger Hairpin (PS1-hp) Structures in Mini-Chromosome Maintenance Proteins of Saccharomyces cerevisiae and Archaea
Source: G3 (Bethesda). 2014 May 23;4(7):1319–26. doi: 10.1534/g3.114.011668 (PMC4455780; doi:10.1534/g3.114.011668)
Supplement: Supporting Information [file supp_4_7_1319__index.html]

Functional Conservation of the Pre-Sensor One Beta-Finger Hairpin (PS1-hp) Structures in Mini-Chromosome Maintenance Proteins of Saccharomyces cerevisiae and Archaea — Supporting Information 

# Functional Conservation of the Pre-Sensor One Beta-Finger Hairpin (PS1-hp) Structures in Mini-Chromosome Maintenance Proteins of *Saccharomyces cerevisiae* and Archaea

## Supporting Information for Ramey and Sclafani, 2014

**Files in this Data Supplement:**

- Supporting Information - Figures S1-S4 (PDF, 710 KB)
- Figure S1 - Protein stability of PS1-hp mutants of Mcm5p and Mcm4p. (PDF, 193 KB)
- Figure S2 - Sensitivity of PS1-hp mutants to replicational stress. (PDF, 292 KB)
- Figure S3 - Time course and flow cytometry analysis of a conditional PS1-hp double mutant. (PDF, 249 KB)
- Figure S4 - Tetrad analysis of PS1-hp mutants and the terminal MCM phenotype of *mcm4 mcm5* PS1-hp double mutant spores. (PDF, 251 KB)
