# Supplementary material for: Functional Conservation of the Pre-Sensor One Beta-Finger Hairpin (PS1-hp) Structures in Mini-Chromosome Maintenance Proteins of Saccharomyces cerevisiae and Archaea
Source: G3 (Bethesda). 2014 May 23;4(7):1319–26. doi: 10.1534/g3.114.011668 (PMC4455780; doi:10.1534/g3.114.011668)
Supplement: Supporting Information [file supp_g3.114.011668_011668SI.pdf]

G3 · Genes | Genomes | Genetics

Functional Conservation of the Pre-Sensor One Beta-finger Hairpin (PS1-hp) Structures in MCM Proteins of *Saccharomyces cerevisiae* and Archaea

Authors: Christopher J. Ramey and Robert A. Sclafani

Department of Biochemistry and Molecular Genetics, University of Colorado Anschutz Medical Campus, Aurora, CO 80045

Corresponding Author: Robert A. Sclafani, 12801 E.17th Ave, MS8101, University of Colorado, Aurora, CO 80045; 303-724-3271; Robert.sclafani@ucdenver.edu

DOI: 10.1534/g3.114.011668

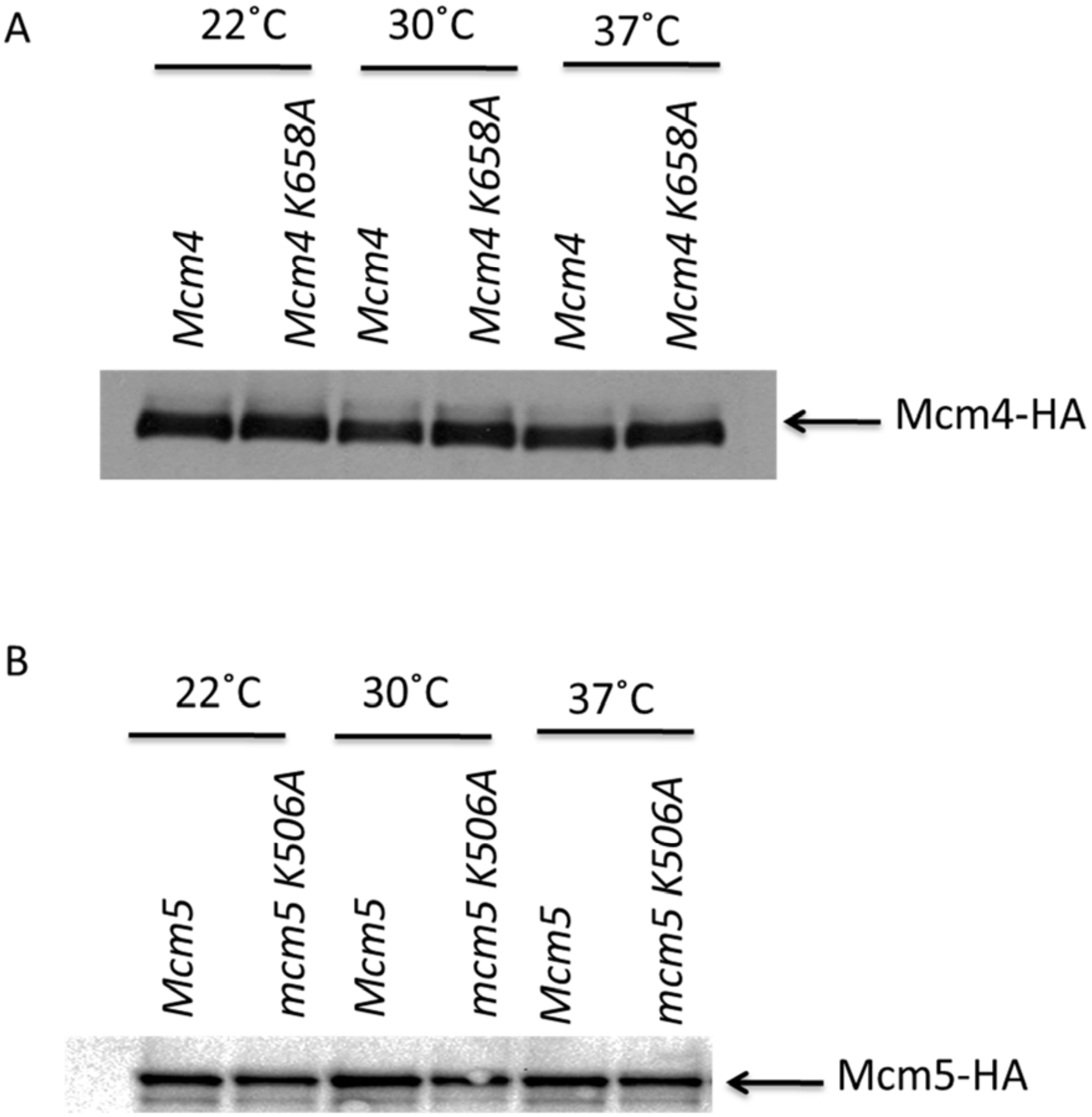

Figure S1 Protein stability of PS1-hp mutants of Mcm5p and Mcm4p. A) Western blot analysis of mcm4p-K658A-HA stability compared to Mcm4p-HA stability at 22˚C, 30˚C and 37˚C. B) Western blot analysis of mcm5p-K506A-HA stability compared to Mcm5p-HA stability at 22˚C, 30˚C and 37˚C.

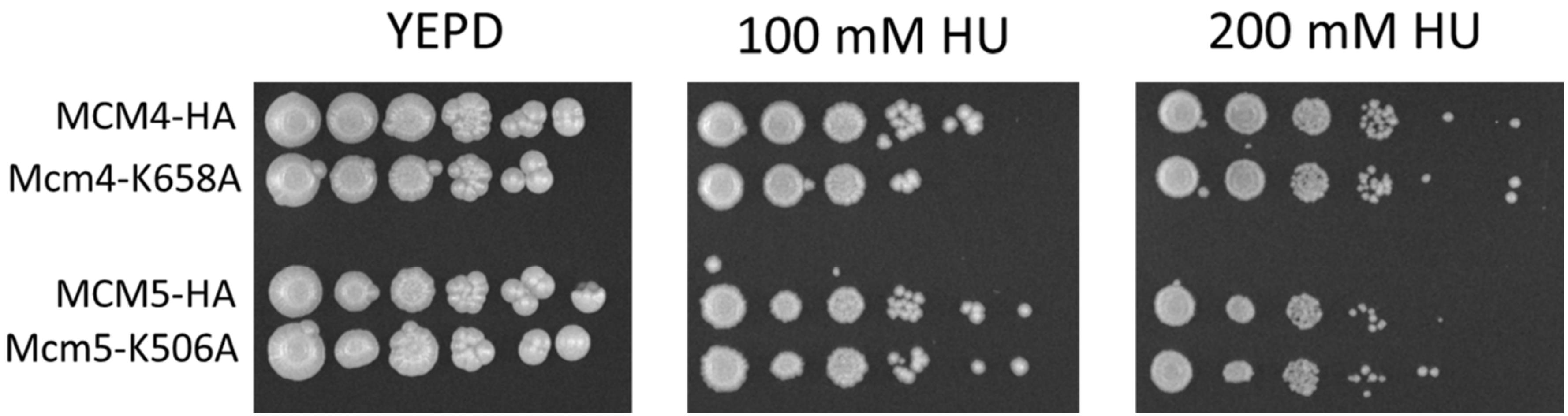

Figure S2 Sensitivity of PS1-hp mutants to replicational stress. A) Serial dilution analysis (10 fold) of growth indicates no growth defect of Mcm4p PS1-hp or Mcm5p PS1-hp mutants in the presence of the replication inhibitor hydroxyurea.

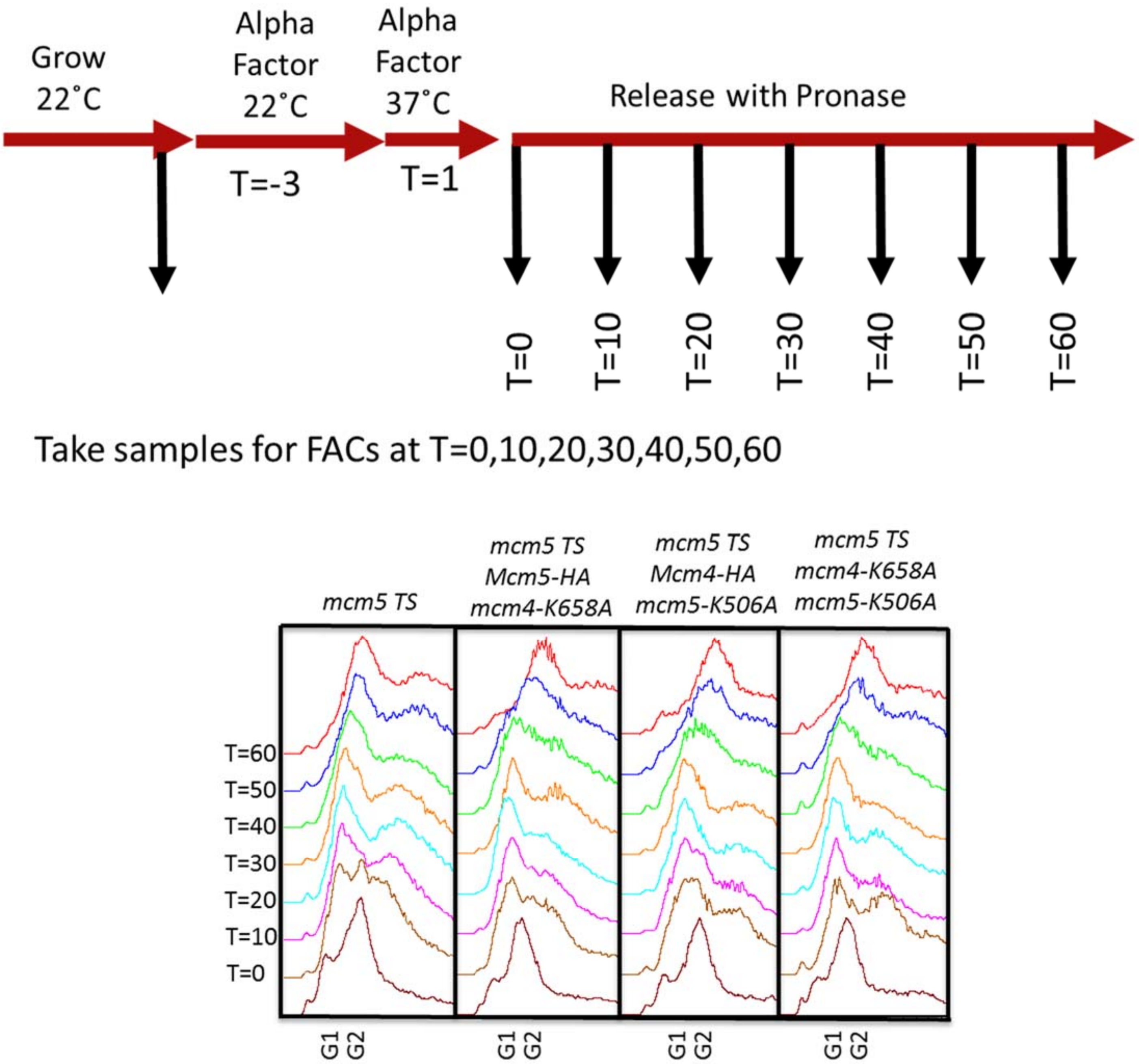

Figure S3 Time course and flow cytometry analysis of a conditional PS1-hp double mutant. RSY1148 (*mcm5-TS*), CRY207 (*mcm5-TS, MCM5-HA::URA3, mcm4-HA K658A::TRP1*), CRY208 (*mcm5-TS, mcm5-HA K506A::URA3, MCM4-HA::TRP1*) and CRY209 (*mcm5-TS, mcm5-HA K506A::URA3, mcm4-HA K658A::TRP1*) strains were arrested in alpha factor at 22˚C for 3 hours then raised to 37˚C for 1 hour. Cells were then released into S Phase at 37˚C by the addition of pronase. Cell cycle progress was followed by flow cytometry. Samples were collected every 10 mins for 60 min then processed and analyzed by FACS.

Synthetic Complete Growth Media (-/+) Indicated Nutrient/Drug

A

Number of Tetrads Dissected	YEPD	-URA	-TRP	-LEU	+ G418	-URA + G418	-URA -TRP	-URA –TRP + G418
91	197	131	88	99	44	44	51	0 (22)

B

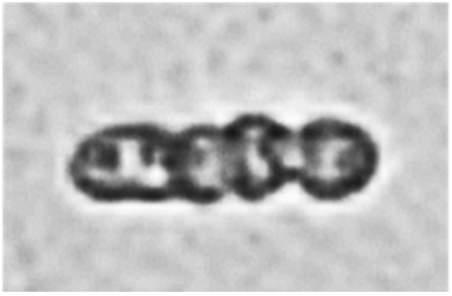

Figure S4 Tetrad analysis of PS1-hp mutants and the terminal MCM phenotype of *mcm4 mcm5* PS1-hp double mutant spores. A. CRY119 was mated to RSY1345 to form diploids, which were then sporulated. Tetrads were dissected and spores that were –Ura$^+$ G418R, which contain both the *mcm5::KanMX4 and mcm5-HAT K658A::URA3* mutations, were tested for the presence of the *mcm4-HA K658A::TRP1* allele. As *mcm5* and *mcm4* are unlinked, we expected to find 50% (22/44) Trp$^+$ colonies that carry the *mcm4* PS1-hp mutation. With *mcm5-HA K658A::URA3,* no Trp$^+$ colonies were found (n=44, p<0.005). **B.** The terminal phenotype of the double *mcm4 mcm5* PS1-hp mutant is two large budded cells (magnification=400X).
