# Supplementary material for: Functional Conservation of the Pre-Sensor One Beta-Finger Hairpin (PS1-hp) Structures in Mini-Chromosome Maintenance Proteins of Saccharomyces cerevisiae and Archaea
Source: G3 (Bethesda). 2014 May 23;4(7):1319–26. doi: 10.1534/g3.114.011668 (PMC4455780; doi:10.1534/g3.114.011668)
Supplement: Supporting Information [file supp_g3.114.011668_FigureS1.pdf]

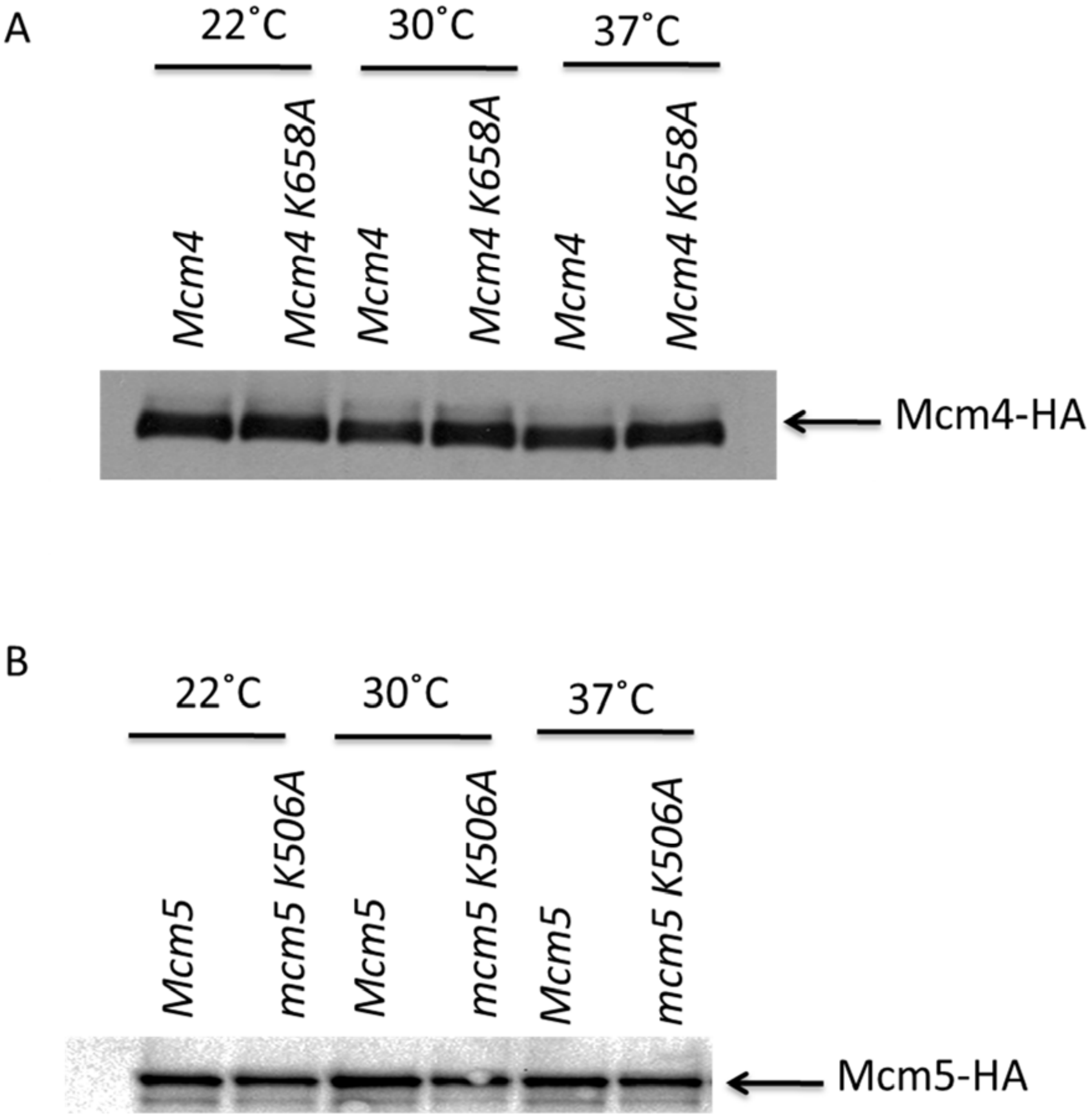

Figure S1 Protein stability of PS1-hp mutants of Mcm5p and Mcm4p. A) Western blot analysis of mcm4p-K658A-HA stability compared to Mcm4p-HA stability at 22˚C, 30˚C and 37˚C. B) Western blot analysis of mcm5p-K506A-HA stability compared to Mcm5p-HA stability at 22˚C, 30˚C and 37˚C.
