# Supplementary material for: Functional Conservation of the Pre-Sensor One Beta-Finger Hairpin (PS1-hp) Structures in Mini-Chromosome Maintenance Proteins of Saccharomyces cerevisiae and Archaea
Source: G3 (Bethesda). 2014 May 23;4(7):1319–26. doi: 10.1534/g3.114.011668 (PMC4455780; doi:10.1534/g3.114.011668)
Supplement: Supporting Information [file supp_g3.114.011668_FigureS2.pdf]

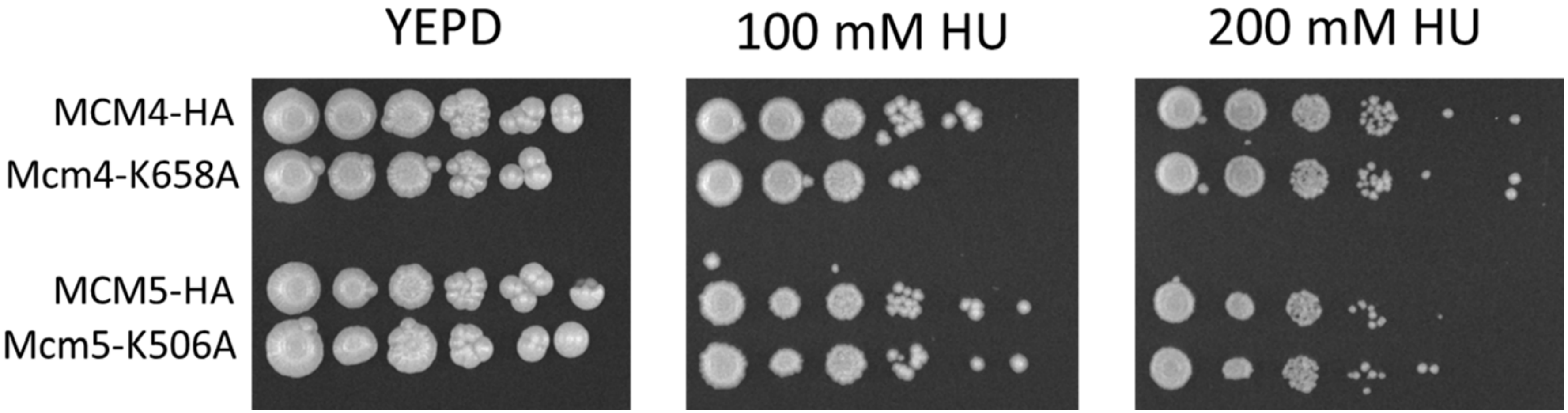

Figure S2 Sensitivity of PS1-hp mutants to replicational stress. A) Serial dilution analysis (10 fold) of growth indicates no growth defect of Mcm4p PS1-hp or Mcm5p PS1-hp mutants in the presence of the replication inhibitor hydroxyurea.
