# Supplementary material for: Functional Conservation of the Pre-Sensor One Beta-Finger Hairpin (PS1-hp) Structures in Mini-Chromosome Maintenance Proteins of Saccharomyces cerevisiae and Archaea
Source: G3 (Bethesda). 2014 May 23;4(7):1319–26. doi: 10.1534/g3.114.011668 (PMC4455780; doi:10.1534/g3.114.011668)
Supplement: Supporting Information [file supp_g3.114.011668_FigureS3.pdf]

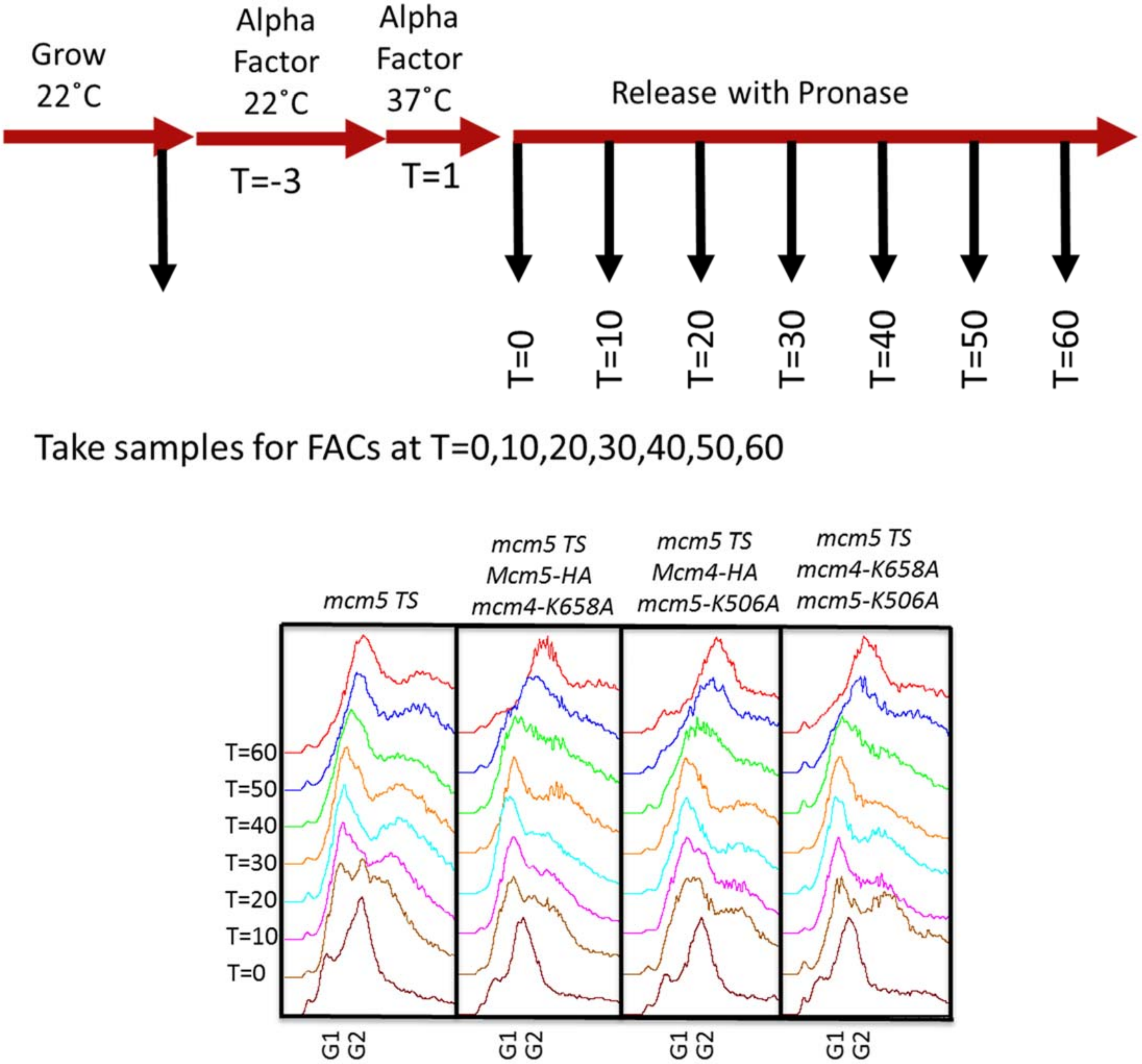

Figure S3 Time course and flow cytometry analysis of a conditional PS1-hp double mutant. RSY1148 (*mcm5-TS*), CRY207 (*mcm5-TS, MCM5-HA::URA3, mcm4-HA K658A::TRP1*), CRY208 (*mcm5-TS, mcm5-HA K506A::URA3, MCM4-HA::TRP1*) and CRY209 (*mcm5-TS, mcm5-HA K506A::URA3, mcm4-HA K658A::TRP1*) strains were arrested in alpha factor at 22˚C for 3 hours then raised to 37˚C for 1 hour. Cells were then released into S Phase at 37˚C by the addition of pronase. Cell cycle progress was followed by flow cytometry. Samples were collected every 10 mins for 60 min then processed and analyzed by FACS.
