# Supplementary material for: Functional Conservation of the Pre-Sensor One Beta-Finger Hairpin (PS1-hp) Structures in Mini-Chromosome Maintenance Proteins of Saccharomyces cerevisiae and Archaea
Source: G3 (Bethesda). 2014 May 23;4(7):1319–26. doi: 10.1534/g3.114.011668 (PMC4455780; doi:10.1534/g3.114.011668)
Supplement: Supporting Information [file supp_g3.114.011668_FigureS4.pdf]

Synthetic Complete Growth Media (-/+) Indicated Nutrient/Drug

A

Number of Tetrads Dissected	YEPD	-URA	-TRP	-LEU	+ G418	-URA + G418	-URA -TRP	-URA –TRP + G418
91	197	131	88	99	44	44	51	0 (22)

B

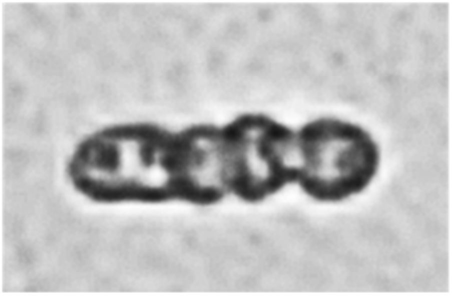

Figure S4 Tetrad analysis of PS1-hp mutants and the terminal MCM phenotype of *mcm4 mcm5* PS1-hp double mutant spores. A. CRY119 was mated to RSY1345 to form diploids, which were then sporulated. Tetrads were dissected and spores that were –Ura^+ G418R, which contain both the *mcm5::KanMX4 and mcm5-HAT K658A::URA3* mutations, were tested for the presence of the *mcm4-HA K658A::TRP1* allele. As *mcm5* and *mcm4* are unlinked, we expected to find 50% (22/44) Trp^+ colonies that carry the *mcm4* PS1-hp mutation. With *mcm5-HA K658A::URA3,* no Trp^+ colonies were found (n=44, p<0.005). **B.** The terminal phenotype of the double *mcm4 mcm5* PS1-hp mutant is two large budded cells (magnification=400X).
